# Intraoperative frozen section analysis of the proximal bile ducts in hilar cholangiocarcinoma is of limited value

**DOI:** 10.1002/cam4.693

**Published:** 2016-04-06

**Authors:** Hendrik T.J. Mantel, Andrie C. Westerkamp, Egbert Sieders, Paul M. J. G. Peeters, Koert P. de Jong, Marieke T. Boer, Ruben H. de Kleine, Annette S. H. Gouw, Robert J. Porte

**Affiliations:** ^1^ Department of Hepato‐Pancreatico‐Biliary Surgery and Liver TransplantationUniversity of GroningenUniversity Medical Center GroningenGroningenThe Netherlands; ^2^ Department of PathologyUniversity of GroningenUniversity Medical Center GroningenGroningenThe Netherlands

**Keywords:** Frozen section analysis, hilar cholangiocarcinoma, Klatskin tumor, prognosis

## Abstract

Frozen section analysis (FS) during cancer surgery is widely used to assess resection margins. However, in hilar cholangiocarcinoma (HCCA), FS may be less reliable because of the specific growth characteristics of the tumor. The aim of this study was to determine the accuracy and consequences of intraoperative FS of the proximal bile duct margins in HCCA. Between 1990 and 2014, 67 patients underwent combined extrahepatic bile duct resection and partial liver resection for HCCA with the use of FS. Sensitivity and specificity of FS was 68% and 97%, respectively. Seventeen of 67 patients (25%) displayed a positive bile duct margin at FS. The false‐negative rate was 16% (eight patients). Ten patients (15%) with a positive bile duct margin underwent an additional resection in an attempt to achieve negative margins, which succeeded in three patients (4%). However, only one of these three patients did not have concomitant lymph node metastases, which are associated with a poor prognosis by itself. The use of FS of the proximal bile duct is of limited clinical value because of the relatively low sensitivity, high risk of false‐negative results, and the low rate of secondary obtained tumor‐free resection margins. Supported by the literature, a new approach to the use of FS in HCCA should be adopted, reserving the technique only for cases in which a substantial additional resection is possible.

## Introduction

Hilar cholangiocarcinoma (HCCA) or Klatskin's disease is a devastating tumor originating from the biliary tract at the confluence of the right and left hepatic duct. Its treatment remains a challenge and the only chance for curation is a radical surgical resection 1. In many cases, the disease has already progressed to advanced stages at presentation and for these patients surgery is not feasible anymore. In contrast, in cases where complete resection is obtained, 5‐year survival rates of 25–40% have been reported 2. For patients with nonradical resections (R1 or R2 status) and/or lymph node metastases the survival is in most cases comparable to the survival of patients without surgical resection 3, 4, 5, 6, 7, 8, 9, 10. In an attempt to achieve radical resection, intraoperative frozen section analysis (FS) of the surgical margins is used to determine whether an additional resection is necessary. This technique is widely used in the surgical treatment of various malignancies including gynecological, pancreatic, liver, and breast cancer 11, 12, 13, 14. However, in the treatment of HCCA the accuracy of FS is potentially compromised because of the specific pathologic features of the tumor. HCCA is often not a bulky tumor, but instead has a branch‐like appearance, spreading longitudinally along the axis of the bile duct wall, partially in the submucosal space with perineural, perilymphatic, and perivascular infiltration 15, 16. This growth pattern may result in increased sampling errors.

Further, the clinical implications of a positive result of intraoperative frozen section analysis are not always straight‐forward. Although a positive bile duct resection margin found at FS should guide toward a more extensive resection, this is not always possible in the surgical treatment of HCCA. In contrast to a positive margin of the distal bile duct, which will result in either a more distal dissection of the common bile duct or even in a pancreatoduodenectomy, a positive proximal bile duct resection margin can put the surgical team in a difficult situation because an additional resection of the liver parenchyma is often accompanied by increased morbidity or is sometimes anatomically not possible.

Only a few studies have addressed the issue of intraoperative frozen section analysis of the proximal bile ducts HCCA with contradictory results 17, 18, 19, 20, 21. The aim of this study was to (1) investigate the accuracy of intraoperative frozen section analysis of the proximal bile ducts in patients with HCCA and (2) to determine the extent to which FS contributes to radical resection of HCCA.

## Materials and Methods

### Patients

Between January 1990 and June 2014, a total of 74 patients underwent extrahepatic bile duct resection combined with partial hepatectomy for HCCA at the University Medical Center Groningen. In seven patients FS was not performed (in one case because Caroli's disease was suspected and in the other cases because a maximum resection was performed and it was concluded that FS would not have any consequences) resulting in a study group of 67 patients. Only patients with adenocarcinoma involving the hepatic duct bifurcation (left, right, and/or common hepatic duct) were included in this study. Follow‐up was continued until June 1st, 2015.

### Diagnostic work‐up

During the entire study period, all patients with (suspected) HCCA were discussed in a multidisciplinary team to evaluate eligibility for surgery.

As described previously, the working diagnosis of HCCA was based on clinical symptoms (i.e., “silent jaundice”) and findings at endoscopic retrograde cholangiopancreaticography (ERCP), magnetic resonance cholangiography (MRC), percutaneous transhepatic cholangiography (PTC), or combinations thereof 9. Preoperative pathological diagnosis by endoscopic brushings or percutaneous fine‐needle aspiration was preferred, but in some cases positive cytology could not be obtained and the diagnosis was based on suspicious radiological characteristics only. In our early experience, either an arteriography or a Doppler ultrasound of the liver vasculature was performed to investigate whether involvement of the portal vein or hepatic artery was present. When high‐resolution computed tomography (CT) angiography became available, four‐phase imaging of the liver was obtained to assess the vascular status. From 2006 magnetic resonance imaging (MRI) and magnetic resonance cholangiopancreaticography (MRCP) was increasingly used to determine the biliary anatomy and progression of the tumor. CT scanning of the thorax was performed to exclude metastases. Diagnostic laparotomy or laparoscopy to rule out peritoneal metastases prior to definitive surgery was not performed.

### Surgical technique

The surgical procedure for resection of HCCA has been described previously 9. Briefly, the procedure started with an explorative laparotomy to exclude any peritoneal metastases. The liver and hepatoduodenal ligament were inspected to determine whether the planned procedure was feasible. If during inspection an enlarged lymph node was encountered at the aortocaval nodal stations or at the celiac trunk (N2 lymph nodes), it was biopsied and sent for frozen section analysis. In case frozen section analysis showed metastatic tumor cells in the sample, the surgical resection was considered futile and the procedure was therefore aborted.

The hepatoduodenal ligament was explored, consisting of regional lymph node dissection and skeletonization of the portal vein and hepatic artery. The distal common bile duct was cut at the level of the pancreatic head. The distal bile duct resection margin was checked with frozen section. In case the distal resection margin was positive, a more distal bile duct transection was performed or in some cases additional pancreaticoduodenectomy.

The bile duct connecting to the future liver remnant was dissected as far as possible into the parenchyma and transected. A frozen section of the proximal bile duct resection margin was taken to be informed about the presence or absence of remnant tumor cells. If a positive margin was encountered, it was evaluated whether a more extensive resection was possible. In some cases, it was concluded that further resection was technically not feasible and the procedure was completed with the knowledge of performing an R1 resection.

### Intraoperative frozen section analysis and final pathology

A 2‐ to 3‐mm segment was taken from the cut ends of bile ducts for frozen section analysis. An imprint was taken from the fresh tissue sample before embedding the margin cuff in Tissue‐Tek^®^ (Sakura Finetek Europe B.V., The Netherlands) and frozen in liquid nitrogen. The 4‐*μ*m thick tissue sections were stained with hemotoxylin–eosin, while the imprint specimen was Giemsa stained. Both specimens were directly analyzed microscopically. Following the intraoperative diagnosis, the frozen samples were fixed in 10% formalin and embedded in paraffin. The 4‐*μ* thick sections were cut from the paraffin blocks and stained with hematoxylin–eosin. The paraffin slides were microscopically examined and the findings were compared with the frozen section results to determine concordancy or discordancy between the two samples. The surgical margin status of the proximal and distal bile ducts was classified as positive when invasive carcinoma or carcinoma in situ was identified microscopically.

An R0 (radical surgical resection) resection was defined as resection without microscopic tumor cells detected in the biliary, vascular, or parenchymal surgical margins. A R1 resection was defined as microscopic tumor deposits in one of the before mentioned surgical margins. A R2 resection was defined as a resection with macroscopic tumor deposits in one or more of the surgical margins. Nodal status was defined positive (N1) if one or more of the hilar lymph nodes contained tumor cells. Patients were staged according to the sixth edition of the American Joint Committee on Cancer (AJCC) TNM staging system for HCCA 22.

### Study design and statistical analysis

Patient data and baseline characteristics were retrospectively collected in a database and statistical analyses were carried out using IBM SPSS Statistics, (IBM, Armonk, New York). Continuous variables were expressed as the means ±SD. Categorical variables were expressed as numbers and percentages. Univariable analyses were conducted for patient survival by Kaplan–Meier estimates of survival probabilities and the log‐rank test for comparisons. *P*‐values of less than 0.05 were considered statistically significant.

Accuracy of intraoperative frozen section analysis of the bile duct margins was determined by calculating the sensitivity, specificity, positive, and negative predictive value. Final histopathological evaluation of the paraffin‐embedded slides was defined as the gold standard.

The consequences of intraoperative frozen section analysis were evaluated in two steps: First, it was assessed whether a positive proximal bile duct resection margin found at FS resulted in an additional resection or not. Second, the final resection status was established at definitive histopathology (radical: R0, or nonradical: R1/R2).

## Results

### Survival and prognostic factors

Patient, operative and final pathology characteristics of the 67 patients who underwent surgical resection for HCCA and frozen section analysis of the proximal bile ducts are presented in Table 1.

**Table 1:** Demographics, surgical characteristics, and final pathological findings in 67 patients with resected hilar cholangiocarcinoma

Variable	Value
Mean age in years (±SD)	61 (±8)
Gender	
Male	40 (60%)
Female	27 (40%)
Liver segments resected in combination with EHBD resection	
1234	22 (32%)
145678	12 (17%)
45678	11 (16%)
5678	8 (12%)
234	5 (8%)
4	4 (6%)
15678	2 (3%)
14	1 (2%)
123	1 (2%)
123458	1 (2%)
Additional pancreatoduodenectomy	6 (9%)
Vascular reconstruction	
No	52 (78%)
Yes	
Venous	11 (16%)
Arterial	2 (3%)
Arterial and venous	2 (3%)
Mean estimated blood loss in mL (±SD)	2200 (±1800)
Tumor stage^a^	
IA	2 (3%)
IB	18 (27%)
IIA	16 (24%)
IIB	29 (43%)
III	0 (0%)
IV	2 (3%)

EHBD, extrahepatic bile duct.

a AJCC TNM staging system (6th edition, 2002).

The 90‐day mortality was 18% (12 patients), ranging from 4 to 80 days. For survival analyses, patients with 90‐day mortality were excluded. The overall 1‐, 3‐, and 5‐year survival rates in the remaining 55 patients was 84%, 46%, and 32%, respectively. Prognostic factors for survival were identified using univariable analyses (Table 2). Final resection status approached significance, but only lymph node status was shown to be a predictive factor for survival.

**Table 2:** Univariable analysis of postoperative survival in patients with hilar cholangiocarcinoma

Variable	No. patients	% 5‐year survival	*P*‐value
Age (year)			
<60	23 (42%)	28	0.55
≥60	32 (58%)	35	
Gender			
Male	33 (60%)	37	0.49
Female	22 (40%)	24	
Type of hepatectomy			
Left	29 (53%)	38	0.39
Right	26 (47%)	24	
pT stage^a^			
T1	2 (4%)	100	0.21
T2	29 (53%)	44	
T3	24 (43%)	13	
Perineural invasion			
Negative	9 (19%)	33	0.91
Positive	39 (81%)	35	
Lymph node metastases			
Negative	31 (56%)	44	0.009
Positive	24 (44%)	17	
Final resection status			
R0	30 (55%)	39	0.054
R1	25 (45%)	22	

Twelve patients with 90‐day mortality were excluded from the analysis.

a According to the sixth edition of the AJCC Cancer Staging Manual.

### Accuracy calculations of frozen section analysis

Accuracy calculations of FS of the proximal bile duct margins are summarized in Table 3. Sensitivity and specificity of intraoperative frozen section analysis of the bile duct margins were 68% and 97%, respectively. The positive predictive value and negative predictive value of intraoperative frozen section analysis of the proximal bile duct margins were both 89%.

**Table 3:** Accuracy calculation of frozen section analysis of the proximal bile duct margin during surgical resection of hilar cholangiocarcinoma^a^

	Positive resection margin at final pathology	Negative resection margin at final pathology	
Positive result intraoperative frozen section analysis	17	2	19
Negative result intraoperative frozen section analysis	8	63	71
	25	65	90
Sensitivity	68%		
Specificity	97%		
Positive likelihood ratio	22		
Positive predictive value	89%		
Negative predictive value	89%		

a In some patients more than one proximal bile duct was examined by frozen section analysis.

### Consequences of frozen section analysis of the proximal bile ducts

**Figure 1:**
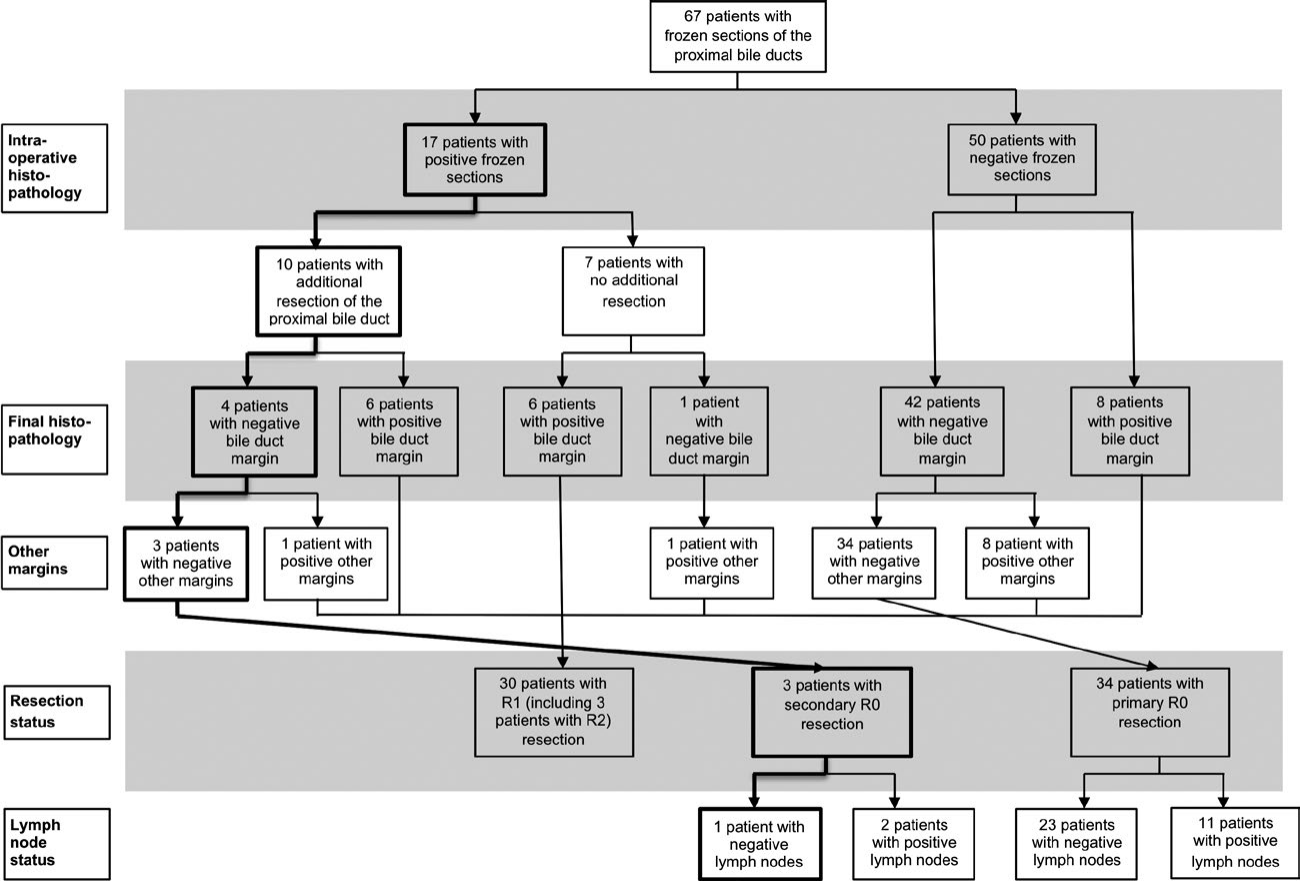
Flow diagram elucidating proximal bile duct margin status at intraoperative and final pathology, additional proximal bile duct resections, other margins’ positivity, final pathology, and defintive lymph node status.

The consequences of FS are visualized in Figure 1. Of the 67 patients in whom frozen section analysis of the proximal bile ducts was performed, 50 patients had a negative result. However, in eight of these patients (16%), FS was false negative and at final histopathology it was found that the proximal bile duct margin was positive for remnant tumor cells (R1 resection).

Seventeen patients (25%) had a positive margin at FS. In 10 patients (15%), an additional resection of the proximal bile duct(s) was performed in an attempt to achieve tumor‐free bile duct margins. In four patients (6%), the additional resections were successful and resulted in secondary tumor‐free bile duct margins. However, final histopathology demonstrated in one of these four patients another positive surgical margin (liver parenchyma).

The length of the additional resections in the three remaining patients was >5 mm. One patient underwent an additional liver segment resection with wide margins and in the other two an additional proximal bile duct resection >5 mm was possible. Two out of three patients in which a secondary R0 resection was achieved, showed concomitant N1 lymph node metastases. Both patients died as a result of tumor recurrence at, respectively, 7 months and two‐and‐a‐half years after surgery. As a consequence, only in one of 67 patients (1.5%) the final resection status and prognosis improved by intraoperative frozen section analysis of the proximal bile ducts. This patient is still alive with a follow‐up of 10 years.

### Recurrence of disease

For recurrence‐of‐disease analyses patients with 90‐day mortality (*n* = 12) were excluded. There were 21 patients with local recurrences and nine patients with distant metastases.

**Figure 2:**
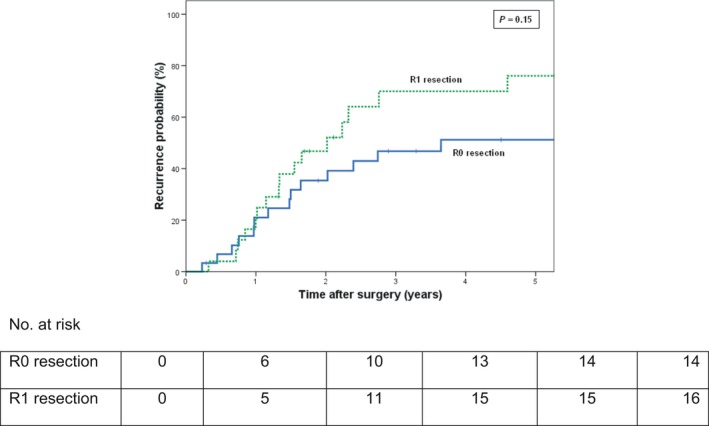
Cumulative probability of reccurence after resection for hilar cholangiocarcinoma according to final resection status (R0 vs. R1). Patients with in‐hospital mortality were excluded from the analysis. *P* = 0.15 (log‐rank test).

Local recurrences were found in 10/30 patients (33%) with an R0 resection versus 11/25 patients (44%) with an R1 resection (*P* = 0.43). To investigate the effect of final resection status on the probability of recurrence, a time‐to‐recurrence analysis was performed. At 5 years follow‐up, the estimated probability of recurrence was 51% in patients with an R0 resection versus 76% in patients with an R1 resection (*P* = 0.15) (Fig. 2). It was concluded that final resection status had no impact on recurrence rate.

## Discussion

This study shows that intraoperative frozen section analysis of the proximal bile ducts is of limited value because of two reasons.

First, we found a low sensitivity rate of 68%, which means that in almost one‐third of cases, FS did not detect tumor cells at the resection margin. In addition, the false‐negative rate of 16% was relatively high, corresponding to eight patients, in whom an additional resection was erroneously withheld, resulting in suboptimal treatment. The best explanation for the impaired sensitivity of intraoperative frozen section analysis in HCCA is the specific growth pattern of the tumor, which is characterized by a longitudinal, infiltrative extension along the mucosa and submucosal spaces leading to sampling errors 15, 16. A few studies are available describing the use of frozen section analysis in the treatment of HCCA (Table 4). Only Okazaki et al. 17 reported a sensitivity rate of 75%, which is comparable to the sensitivity rate of 68% found in this study. However, most studies did specify their rate of false‐negative results, ranging from 2 to 28. 17, 18, 19, 20 The false‐negative rate of 16% in our series is therefore in accordance with the literature.

**Table 4:** Overview of the literature

Author	n	Sensitivity	Number of patients in which a secondary R0 resection was obtained	Survival benefit of secondary R0 resections compared to R1 resection?
Okazaki et al., 2002 17	23	75%	0 (0%)	–
Endo et al., 2008 18	101	NR	9 (9%)	no
Shingu et al., 2009 19	138	NR	8 (6%)	no
Ribero et al., 2011 21	67	NR	13 (19%)	yes
Lee et al., 2012 20	162	NR	7 (4%)	no
Present study	67	68%	3 (4%)	–

NR: not reported.

Second, the extent to which FS contributed to radical resections was low, because in only three patients a secondary R0 resection could be achieved. In all other cases, additional resection was not possible or failed in its goal to obtain a negative bile duct margin. Table 4 shows that low rates of secondary obtained R0 resections (4–9%) are reported by most authors. 18, 19, 20 In many cases, additional resection guided by a positive result of frozen section analysis is not possible from anatomic and technical points of view. An exception to the rule is the article from Ribero et al. 21 which showed a secondary R0 resection rate of 19%. A possible explanation for this difference is that these authors performed relatively minor resections as was suggested by one of the discussants at the end of the article. However, the distribution of (extended) left and (extended) right hepatectomy was very comparable to our series. It should be noted that the high rate of secondary obtained R0 resections in this study was accompanied by a high rate of postoperative complications, especially biliary fistula, suggesting that additional resections are indeed technically demanding 21.

An important question is whether or not a secondary R0 resection will improve prognosis. Unfortunately, our numbers were not large enough to allow a survival analysis comparing patients with a secondary obtained R0 resection to those with an R1 or primary R0 resection. Table 4 shows that four authors addressed this issue. Endo et al. 18 and Shingu et al. 19 were able to perform a formal survival analysis and found that secondary obtained R0 resections did not improve prognosis. Survival in the secondary R0 group was comparable to the group with R1 resections. Lee et al. 20 did not perform a survival analysis, but noticed disease recurrence and subsequent tumor‐induced death in five of seven patients with a secondary obtained R0 resections. These authors also concluded that there was no survival benefit of secondary achieved radical resections. Ribero et al. 21 published the only study in which an improvement in prognosis was found in the secondary R0 group. Thirteen patients in this group had a 5‐year survival rate of 50% which was comparable to the primary R0 group (5‐year survival rate of 31%) and significantly better than the R1 group (5‐year survival rate of 0%).

In the present study, lymph node status was deliberately included in the overview of the final pathology. The presence of regional lymph node metastases has repeatedly been shown to have a negative impact on survival 4, 23, 24, which was also confirmed in our univariable analysis (Table 2). When brought into consideration, regional lymph node metastases were found in two of three patients with a secondary obtained R0 resection, which is detrimental to the expected prognosis. In retrospect, it can be concluded that of 67 patients in which frozen section analysis was performed, only one patient ultimately benefitted from the procedure because in this patient resection margins as well as lymph node status were negative.

Considering the low sensitivity rate, high false‐negative rate, and low rate of secondary achieved R0 resections in our series, together with the lack of improved survival after secondary R0 resection reported by most authors in the literature, it could be concluded that frozen section analysis has no value in the treatment of HCCA. Some authors have advocated performing extended resections as the surgical procedure of choice since it results in the highest rates of R0 resections and 5‐year survival. 25 When adopting this strategy, frozen section analysis of the proximal bile duct can indeed be abandoned because further resection will be impossible. However, an interesting observation was made by Endo et al. 18 that patients with a wide resection margin of the proximal bile duct showed a significant better 5‐year survival than patients with a narrow resection margin, 43% versus 18%, respectively. It therefore seems reasonable to not completely abandon the use of frozen section analysis of the proximal bile ducts, but to reserve this technique only for those cases in which an additional resection with substantial length is possible. The length of the additional resection could be estimated on >5 mm since a tumor‐free proximal bile duct margin of 5 mm was found to be sufficient in avoiding local recurrences 26.

Our study has a number of disadvantages related to its retrospective design and limited number of patients, which prevented us from performing a survival analysis of the secondary R0 group. However, as can be concluded from Table 4, limited numbers are common in most studies of HCCA since hilar cholangiocarcinoma is a rare disease.

## Conclusions

In conclusion, this study shows that the use of intraoperative frozen section analysis of the proximal bile ducts has a low sensitivity, high rate of false‐negative results, and a limited contribution in obtaining secondary R0 resections. This finding, together with the fact that secondary R0 resections do not improve survival in most reports in the literature, justifies a new approach to the use of FS, reserving the technique only for those cases in which a substantial additional resection is possible.

## Conflict of Interest

The authors declare that they have nothing to disclose.

## References

[1] Khan, S. A. , H. C. Thomas , B. R. Davidson , and S. D. Taylor‐Robinson . 2005 Cholangiocarcinoma. Lancet 366:1303–1314. 16214602 10.1016/S0140-6736(05)67530-7

[2] Ito, F. , C. S. Cho , L. F. Rikkers , and S. M. Weber . 2009 Hilar cholangiocarcinoma: current management. Ann. Surg. 250:210–218. 19638920 10.1097/SLA.0b013e3181afe0ab

[3] Dinant, S. , M. F. Gerhards , E. A. Rauws , O. R. Busch , D. J. Gouma , T. M. van Gulik , et al. 2006 Improved outcome of resection of hilar cholangiocarcinoma (Klatskin tumor). Ann. Surg. Oncol. 13:872–880. 16614876 10.1245/ASO.2006.05.053

[4] DeOliveira, M. L. , S. C. Cunningham , J. L. Cameron , F. Kamangar , J. M. Winter , K. D. Lillemoe , et al. 2007 Cholangiocarcinoma: thirty‐one‐year experience with 564 patients at a single institution. Ann. Surg. 245:755–762. 17457168 10.1097/01.sla.0000251366.62632.d3 PMC1877058

[5] Ramacciato, G. , G. Nigri , R. Bellagamba , N. Petrucciani , M. Ravaioli , M. Cescon , et al. 2010 Univariate and multivariate analysis of prognostic factors in the surgical treatment of hilar cholangiocarcinoma. Am. Surg. 76:1260–1268. 21140696

[6] Nuzzo, G. , F. Giuliante , F. Ardito , A. M. De Rose , M. Vellone , G. Clemente , et al. 2010 Intrahepatic cholangiocarcinoma: prognostic factors after liver resection. Updates Surg. 62:11–19. 20845096 10.1007/s13304-010-0007-x

[7] Rocha, F. G. , K. Matsuo , L. H. Blumgart , and W. R. Jarnagin . 2010 Hilar cholangiocarcinoma: the Memorial Sloan‐Kettering Cancer Center experience. J. Hepatobiliary Pancreat. Sci. 17:490–496. 19806295 10.1007/s00534-009-0205-4

[8] Matsuo, K. , F. G. Rocha , K. Ito , M. I. D'Angelica , P. J. Allen , Y. Fong , et al. 2012 The blumgart preoperative staging system for hilar cholangiocarcinoma: analysis of resectability and outcomes in 380 patients. J. Am. Coll. Surg. 215:343–355. 22749003 10.1016/j.jamcollsurg.2012.05.025

[9] IJitsma, A. J. , B. M. Appeltans , K. P. de Jong , R. J. Porte , P. M. Peeters , and M. J. Slooff . 2004 Extrahepatic bile duct resection in combination with liver resection for hilar cholangiocarcinoma: a report of 42 cases. J. Gastrointest. Surg. 8:686–694. 15358329 10.1016/j.gassur.2004.04.006

[10] Khan, S. A. , B. R. Davidson , R. D. Goldin , N. Heaton , J. Karani , S. P. Pereira , et al. 2012 Guidelines for the diagnosis and treatment of cholangiocarcinoma: an update. Gut 61:1657–1669. 22895392 10.1136/gutjnl-2011-301748

[11] Tangjitgamol, S. , S. Jesadapatrakul , S. Manusirivithaya , and C. Sheanakul . 2004 Accuracy of frozen section in diagnosis of ovarian mass. Int. J. Gynecol. Cancer 14:212–219. 15086718 10.1111/j.1048-891X.2004.014202.x

[12] Cioc, A. M. , E. C. Ellison , D. M. Proca , J. G. Lucas , and W. L. Frankel . 2002 Frozen section diagnosis of pancreatic lesions. Arch. Pathol. Lab. Med. 126:1169–1173. 12296752 10.5858/2002-126-1169-FSDOPL

[13] Rakha, E. , S. Ramaiah , and A. McGregor . 2006 Accuracy of frozen section in the diagnosis of liver mass lesions. J. Clin. Pathol. 59:352–354. 16489181 10.1136/jcp.2005.029538 PMC1860366

[14] Ferreiro, J. A. , J. J. Gisvold , and D. G. Bostwick . 1995 Accuracy of frozen‐section diagnosis of mammographically directed breast biopsies. Results of 1490 consecutive cases. Am. J. Surg. Pathol. 19:1267–1271. 7573688 10.1097/00000478-199511000-00006

[15] Bosma, A. 1990 Surgical pathology of cholangiocarcinoma of the liver hilus (Klatskin tumor). Semin. Liver Dis. 10:85–90. 2162569 10.1055/s-2008-1040460

[16] Lim, J. H. , and C. K. Park . 2004 Pathology of cholangiocarcinoma. Abdom. Imaging 29:540–547. 15383897 10.1007/s00261-004-0187-2

[17] Okazaki, Y. , T. Horimi , M. Kotaka , S. Morita , and M. Takasaki . 2002 Study of the intrahepatic surgical margin of hilar bile duct carcinoma. Hepatogastroenterology 49:625–627. 12063955

[18] Endo, I. , M. G. House , D. S. Klimstra , M. Gönen , M. D'Angelica , R. P. Dematteo , et al. 2008 Clinical significance of intraoperative bile duct margin assessment for hilar cholangiocarcinoma. Ann. Surg. Oncol. 15:2104–2112. 18543039 10.1245/s10434-008-0003-2

[19] Shingu, Y. , T. Ebata , H. Nishio , T. Igami , Y. Shimoyama , and M. Nagino . 2010 Clinical value of additional resection of a margin‐positive proximal bile duct in hilar cholangiocarcinoma. Surgery 147:49–56. 19767048 10.1016/j.surg.2009.06.030

[20] Lee, J. H. , D. W. Hwang , S. Y. Lee , K. M. Park , and Y. J. Lee . 2012 The proximal margin of resected hilar cholangiocarcinoma: the effect of microscopic positive margin on long‐term survival. Am. Surg. 78:471–477. 22472407

[21] Ribero, D. , M. Amisano , R. Lo Tesoriere , S. Rosso , A. Ferrero , and L. Capussotti . 2011 Additional resection of an intraoperative margin‐positive proximal bile duct improves survival in patients with hilar cholangiocarcinoma. Ann. Surg. 254:776–781; discussion 781‐3. 22042470 10.1097/SLA.0b013e3182368f85

[22] Green, F. L. 2002 American Joint Committee on Cancer: AJCC Cancer Staging Manual, 6th ed. Springer, New York.

[23] Nimura, Y. , J. Kamiya , S. Kondo , M. Nagino , K. Uesaka , and K. Oda . 2000 Aggressive preoperative management and extended surgery for hilar cholangiocarcinoma: Nagoya experience. J. Hepatobiliary Pancreat. Surg. 7:155–162. 10982608 10.1007/s005340050170

[24] Li, H. , Y. Qin , Y. Cui , H. Chen , X. Hao , and Q. Li . 2011 Analysis of the surgical outcome and prognostic factors for hilar cholangiocarcinoma: a Chinese experience. Dig. Surg. 28:226–231. 21540611 10.1159/000327361

[25] Neuhaus, P. , S. Jonas , W. O. Bechstein , R. Lohmann , C. Radke , N. Kling , et al. 1999 Extended resections for hilar cholangiocarcinoma. Ann. Surg. 230:808–818; discussion 819. 10615936 10.1097/00000658-199912000-00010 PMC1420945

[26] Sakamoto, E. , Y. Nimura , N. Hayakawa , J. Kamiya , S. Kondo , M. Nagino , et al. 1998 The pattern of infiltration at the proximal border of hilar bile duct carcinoma: a histologic analysis of 62 resected cases. Ann. Surg. 227:405–411. 9527064 10.1097/00000658-199803000-00013 PMC1191279

